# Factors contributing to nonalcoholic fatty liver disease (NAFLD) and fat deposition after pancreaticoduodenectomy: A retrospective analysis

**DOI:** 10.1002/ags3.12673

**Published:** 2023-03-28

**Authors:** Hideki Izumi, Hisamichi Yoshii, Rika Fujino, Shigeya Takeo, Eiji Nomura, Masaya Mukai, Hiroyasu Makuuchi

**Affiliations:** ^1^ Department of Gastrointestinal Surgery Tokai University Hachioji Hospital Hachioji, Tokyo Japan

**Keywords:** fatty deposition, nonalcoholic fatty liver disease, pancreatic exocrine insufficiency, pancreaticoduodenectomy

## Abstract

**Aim:**

Nonalcoholic fatty liver disease (NAFLD) can occur due to various reasons after pancreaticoduodenectomy (PD). This study examined the risk and perioperative determinants of NAFLD and fat deposition after PD.

**Methods:**

A total of 101 patients who had undergone computed tomography 6 months after PD were included. We compared perioperative factors between patients who developed NAFLD and those who developed fatty deposits after PD.

**Results:**

In the NAFLD group, pancreatic cancer was significantly more prevalent among patients who developed postoperative NAFLD (*p* = 0.024) and had a lower postoperative body mass index (BMI; *p* = 0.008). Multivariate analysis revealed that pancreatic carcinoma (hazard ratio [HR] 4.42, 95% confidence interval [CI] 1.118–17.442, *p* = 0.034) and lower postoperative BMI (HR 0.51, 95% CI 0.274–0.954, *p* = 0.0355) were risk factors for fatty liver. Pancreatic leakage (*p* = 0.024) and postoperative BMI (*p* = 0.002) were significantly lower in the fat deposition group than those in the NAFLD group. Multivariate analysis also revealed that a lower postoperative BMI was a risk factor for fat deposition (HR 0.56, 95% CI 0.523–0.982, *p* = 0.042). Moreover, multivariate analysis revealed that the fat deposition group had significantly lower pancreatic leakage than the NAFLD group (HR 7.944, 95% CI 1.993–63.562, *p* = 0.049).

**Conclusion:**

The findings of this study suggest that postoperative BMI and pancreatic cancer are associated with a higher risk of NAFLD after PD, possibly because of pancreatic exocrine insufficiency and impaired fat absorption.

## Graphical abstract

Study of factors contributing to fatty liver and fat deposition after pancreaticoduodenectomy.

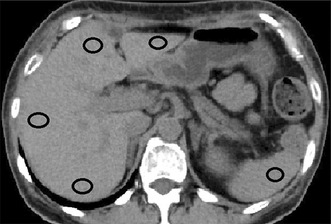

## 1 INTRODUCTION

Nonalcoholic fatty liver disease (NAFLD) is a pathological manifestation of fatty liver disease in the absence of a clear history of alcohol consumption. It is broadly classified into progressive nonalcoholic steatohepatitis (NASH) and nonalcoholic fatty liver (NAFL), with the latter showing little or no disease progression.^1^ In 1980, Ludwig et al.^2^ defined NASH as a set of pathological findings similar to those of alcoholic hepatitis that steadily progress from cirrhosis to liver failure despite the absence of alcohol use. The development of NAFLD associated with lifestyle‐related diseases, such as dyslipidemia and diabetes mellitus, is influenced by environmental and genetic factors.^3^ After gastrointestinal surgery, hyponutrition and various metabolic abnormalities may occur, possibly leading to the development of secondary NAFLD. After total gastrectomy, physiological digestive processes in the stomach are lost, and digestive and absorption disorders may develop due to the lack of gastric acid and pepsin, decreased secretion of gastrointestinal hormones, and impaired pancreatic exocrine secretion.^4^

NAFLD can occur after pancreaticoduodenectomy (PD) at a reported frequency of 15%–40% due to various reasons.^5^, ^6^, ^7^, ^8^ Various risk factors have been reported for the development of NAFLD after PD, including pancreatic cancer, high preoperative HbA1c and CA19‐9 levels, pancreas resection volume, pancreatic stiffness, female sex, pancreatic leakage, postoperative pancreatic exocrine insufficiency, and postoperative impaired intake and diarrhea.^6^, ^7^, ^9^, ^10^, ^11^, ^12^ To the best of our knowledge, no studies have investigated the risk factors for fat deposition after PD, which may precede but not necessarily lead to NAFLD. In this study, we examined the risk and perioperative determinants of NAFLD and fat deposition after PD at our institution.

## 2 METHODS

### 2.1 Patients

In total, 152 PDs were performed at Tokai University Hachioji Hospital between April 2016 and March 2022. Fifteen patients who had not undergone surgery 6 months prior to PD were excluded. In addition, patients who were unable to undergo computed tomography (CT) after 6 months of PD were excluded from the study for the following reasons: benign disease, 16 cases (intraductal papillary mucinous adenoma [IPMA], 14 cases; pancreatitis, two cases); transfer to another hospital, four cases; death from another disease, two cases (one case of intracranial hemorrhage and one case of myocardial infarction); mortality, two cases (one case of hemorrhage and one case of pneumonia); early recurrence, five cases; and patients who did not undergo CT at 6 months of PD, seven cases. Finally, 101 patients who had undergone CT 6 months after PD were included. Informed consent for this retrospective analysis was obtained via the opt‐out method. This study was approved by the institutional review board of Tokai University in November 2022 (Approval Number: 22R‐186).

### 2.2 Surgical procedures

The surgical procedure performed for all patients was subtotal stomach‐preserving PD (SSPPD) via the modified Child method.^13^ Pancreatic duct jejunal mucosal anastomosis was performed. No pancreatic duct stent was placed in patients with main pancreatic duct dilatation. For patients with a soft pancreas without main pancreatic duct dilation, a 5‐Fr pancreatic duct stent (Sumitomo Bakelite) was placed in the jejunum as a lost stent. No external stent was used for pancreatoenterostomy. No external bile fistulas were found, and no jejunostomy or gastrointestinal tube was required. Only one patient required the placement of a closed suction drain from the left side to the dorsal pancreatic jejunal anastomosis. Drinking water and oral nutrition were initiated on the first postoperative day, whereas food intake was initiated on the third postoperative day. The drain was removed after checking it, and the amylase level on the third postoperative day was measured. If no postoperative problems developed, the patient was discharged on the seventh postoperative day. No prescriptions for pancreatic enzyme supplementation, such as LapaCreon® (Eisai Co., Ltd.) or Berizym® (Shionogi & Co., Ltd.), were required.

### 2.3 Definition of NAFLD

**FIGURE 1:**
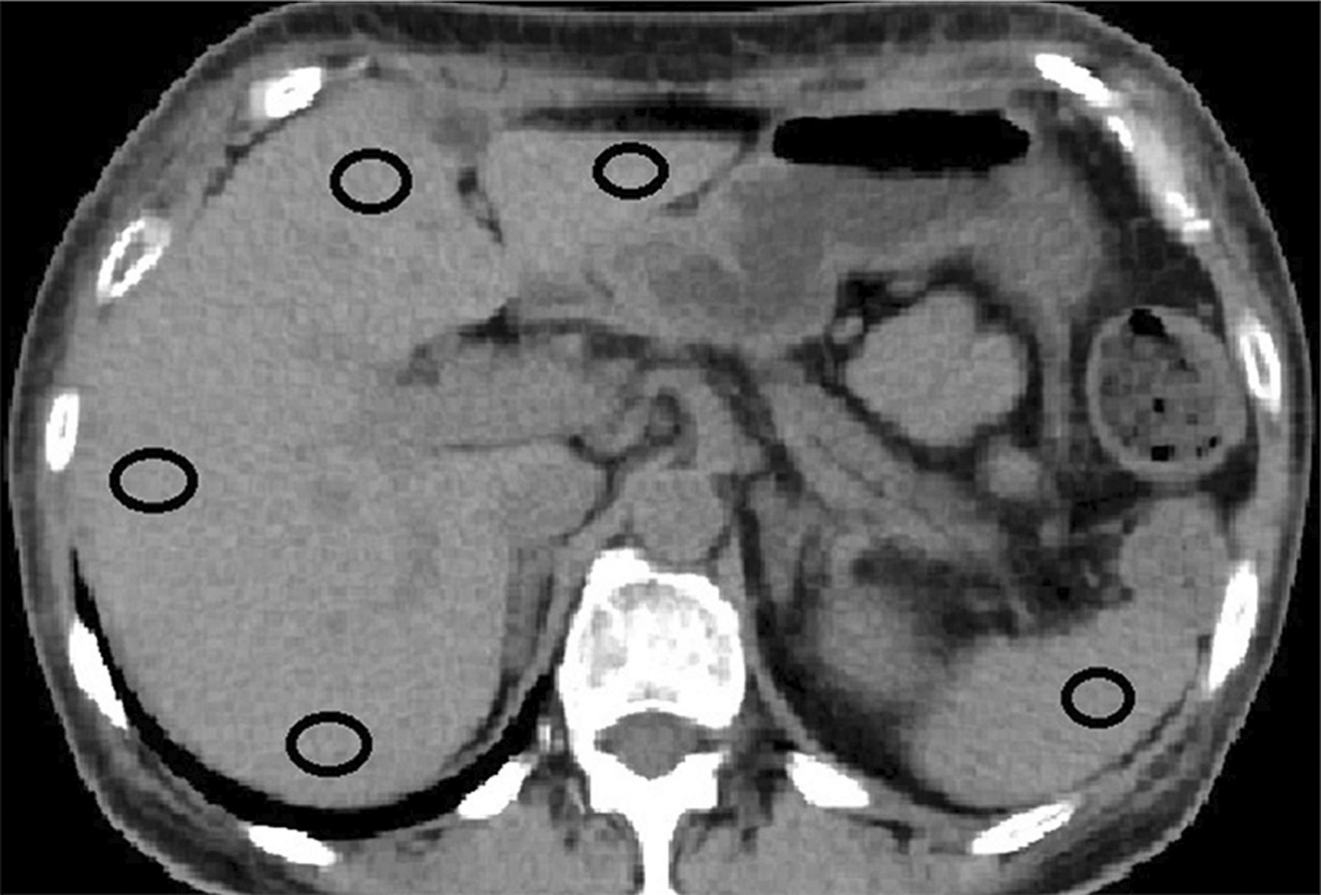
Liver and spleen attenuation values were measured on plain computed tomography images. The degree of liver attenuation was measured in four regions of interest in different sections of the organ. The degree of spleen attenuation was measured in one region of interest in the organ.

The presence of NAFLD was determined through plain CT performed 6 months after PD. Liver and spleen attenuation values were measured on unenhanced CT images and presented in Hounsfield units to quantify the development of NAFLD. Each region of interest (ROI) was a round area of 1.0 cm^2^. We considered the mean value of four ROIs at different sections of the liver as a measure of the degree of liver attenuation (Figure 1). To measure spleen attenuation, a single ROI was used, and NAFLD was defined as a liver‐to‐spleen attenuation ratio^14^ of <0.9.

Next, we defined fat deposition as the stage preceding NAFLD as follows. The difference between the preoperative liver‐to‐spleen attenuation ratio and the 6‐month postoperative liver‐to‐spleen attenuation ratio was calculated. Cases with a difference of 0.10 or more were considered to have a tendency toward fat deposition and were classified into the fat deposition group.

### 2.4 Evaluation of clinical factors

We evaluated variables from the preoperative, intraoperative, and postoperative factors. Preoperative factors included age, sex, diagnosis, albumin content, total protein content, HbA1c level, and body mass index (BMI). Intraoperative factors included surgery duration, blood loss, and surgical procedure. Postoperative factors included albumin content, total protein content, HbA1c level, BMI, postoperative complications, time‐lapse drain removal (days), and length of hospital stay. The 2016 edition of the International Study Group on Pancreatic Surgery (ISGPF) grading system^15^ was used to categorize postoperative pancreatic fistulas (POPFs).

### 2.5 Statistical analysis

All statistical analyses were performed using SPSS version 26.0 for Windows (IBM). Continuous variables were expressed as mean ± standard deviation (SD). Statistically significant differences were determined using the paired *t*‐test or Mann–Whitney *U*‐test. Categorical variables were analyzed using the χ^2^ test or Fisher's test, as appropriate. Risk factors associated with the development of NAFLD and pre‐NAFLD were analyzed using univariate and multivariate analyses. Results were considered statistically significant at a *p* value of <0.05.

## 3 RESULTS

### 3.1 Background of the enrolled patients

The total number of patients enrolled herein was 101. The male‐to‐female ratio was 57:44; the median age was 72 years (interquartile range: 35–87 years); the median surgery duration was 233.0 min (123–507 min); the median blood loss was 631.0 mL (54–3146 mL); and the median postoperative hospital stay was 12.0 days (6–59 days). Fourteen patients (13.9%) had ISGPF Grade B POPF. All patients were able to start drinking water the day after surgery, and all patients were able to start eating on the third postoperative day. The surgical procedures performed were SSPPD in 83 cases, SSPPD + portal vein resection in nine cases, SSPPD + hepatectomy in one case, PD in two cases, and laparoscopic SSPPD in six cases. Among the enrolled patients, there were 45 cases of pancreatic cancer, 26 cases of distal bile duct cancer, 10 cases of intraductal papillary mucinous carcinoma, eight cases of IPMA, and six cases of papillary carcinoma of Vater's papilla. Complications included 14 cases of ISGPF Grade B POPF, eight cases of bile leakage, and seven cases of surgical site infection (SSI) (Table 1).

**TABLE 1:** Background characteristics of the enrolled patients.

Sex (male: Female)	57:44
Age (year), mean (SD)	71.9 (9.2)
Surgery duration (min), mean (SD)	231.0 (69)
Blood loss volume (ml), mean (SD)	631 (525.9)
Diagnosis	
Pancreatic carcinoma	45 (44.6%)
Bile duct carcinoma	26 (25.7%)
IPMC	10 (10.9%)
IPMA	8 (7.9%)
Vater's papilla carcinoma	6 (5.9%)
Duodenal GIST	2 (2.0%)
SCN	1 (1.0%)
Pancreatitis	1 (1.0%)
Pancreatic NET	1 (1.0%)
SPN	1 (1.0%)
Surgical procedure	
SSPPD	83 (82.2%)
SSPPD + PV	9 (8.9%)
Lap‐SSPPD	6 (5.9%)
PD	2 (2.0%)
SSPPD + hepatectomy	1 (1.0%)
Morbidity	
POPF > Grade B	14 (13.9%)
Bile leakage	8 (7.9%)
SSI	2 (2.0%)
Postoperative bleeding	1 (1.0%)
DGE	1 (1.0%)
Pneumonia	1 (1.0%)
Anastomotic stenosis	1 (1.0%)
Hospital stay (days), mean (SD)	12.0 (7.9)

Abbreviations: DGE, delayed gastric emptying; GIST, gastrointestinal stromal tumor; IPMA, intraductal papillary mucinous adenoma; IPMC, intraductal papillary mucinous carcinoma; Lap‐SSPPD, laparoscopic SSPPD; NET, neuroendocrine tumor; PD, pancreaticoduodenectomy; POPF, postoperative pancreatic fistula; PV, portal vein resection; SCN, serous cystic neoplasm; SD, standard deviation; SPT, solid pseudopapillary tumor; SSI, surgical site infection; SSPPD, subtotal stomach‐preserving pancreaticoduodenectomy.

### 3.2 Examination for NAFLD

Only one patient had NAFLD preoperatively. Six months after surgery, NAFLD was identified in 12 patients (11.9%), only one of whom had preoperative NAFLD. The male‐to‐female ratio was 1:2, and the diseases were pancreatic cancer in 10 cases, cholangiocarcinoma in one case, and serous cyst neoplasm in one case. Pancreatic cancer was significantly more prevalent among patients with postoperative NAFLD (*p* = 0.024). SSPPD was performed in 11 cases and PD in one case. Complications included bile leakage in two cases, anastomotic stenosis in one case, and SSI in one case. There was no pancreatic leakage in the NAFLD group; however, the difference in the incidence of this complication between the NAFLD and non‐NAFLD groups was not statistically significant (*p* = 0.149). The time‐lapse until drain removal was shorter in the NAFLD group (5.8 days) than in the non‐NAFLD group (9.9 days); however, the difference in this parameter between the two groups was not statistically significant (*p* = 0.295). No significant differences in blood loss volume, surgery duration, and diagnosis were observed between the NAFLD and non‐NAFLD groups. The NAFLD group had a lower postoperative BMI than the non‐NAFLD group (18.3 vs. 20.9, *p* = 0.008). Multivariate analysis revealed that pancreatic carcinoma (hazard ratio [HR] 4.417, 95% confidence interval [CI] 1.118–17.442, *p* = 0.034) and lower postoperative BMI (HR 0.511, 95% CI: 0.274–0.954, *p* = 0.035) were significantly associated with the risk of NAFLD (Table 2).

**TABLE 2:** Comparison of clinical data between the NAFLD and non‐NAFLD groups.

	NAFLD (*n* = 12)	Non‐NAFLD (*n* = 89)	Univariate analysis	Multivariate analysis		
			*p* value	HR	95% CI	*p* value
Sex (male: female)	4:8	53:35	0.125			NS
Age (year), mean (SD)	71.6 (14.0)	72.7 (8.0)	0.35			NS
Surgery duration (min), mean (SD)	204.4 (61.6)	236.3 (71.0)	0.142			NS
Blood loss volume (mL), mean (SD)	559.8 (424.8)	640.6 (538.1)	0.621			NS
Diagnosis						
Pancreatic carcinoma: Other cancers	10:2	36:53	0.024	4.42	1.118–17.442	0.034
Preoperative albumin (g/dL), mean (SD)	3.6 (0.5)	3.7 (0.5)	0.606			NS
Preoperative total protein (g/dL), mean (SD)	7.1 (0.8)	7.1 (0.8)	0.962			NS
Preoperative HbA1c (%), mean (SD)	7.1 (1.0)	6.4 (1.2)	0.187			NS
Preoperative BMI, mean (SD)	22 (3.6)	22.4 (3.00)	1.647			NS
Surgical procedure						
SSPPD	11	72	0.58			NS
SSPPD + PV	0	9	0.72			NS
Lap‐SSPPD	0	6	0.542			NS
PD	1	1	0.244			NS
SSPPD + hepatectomy	0	1	0.488			NS
Postoperative albumin (g/dL), mean (SD)	3.4 (0.6)	3.6 (0.7)	0.195			NS
Postoperative total protein (g/dL), mean (SD)	7 (0.5)	7.1 (0.7)	0.934			NS
Postoperative HbA1c (%), mean (SD)	6.1 (0.6)	6.2 (0.7)	0.836			NS
Postoperative BMI, mean (SD)	18.3 (2.9)	20.9 (2.6)	0.008	0.51	0.274–0.954	0.035
Complications						
POPF ≥ Grade B	0	14	0.149			NS
Bile leakage	2	6	0.462			NS
Postoperative bleeding	0	1	0.566			NS
SSI	1	1	0.485			NS
Pneumonia	0	1	0.566			NS
Anastomotic stenosis	1	0	0.478			NS
Days until drain removal, mean (SD)	5.8 (6.4)	9.9 (12.9)	0.295			NS
Hospital stay (days), mean (SD)	15 (14.6)	11.6 (6.3)	0.452			NS

Abbreviations: BMI, body mass index; CI, confidence interval; HR, hazard ratio; Lap‐SSPPD, laparoscopic SSPPD; NAFLD, nonalcoholic fatty liver disease; NS, not significant; PD, pancreaticoduodenectomy; POPF, postoperative pancreatic fistula; PV, portal vein resection; SD, standard deviation; SSI, surgical site infection; SSPPD, subtotal stomach‐preserving pancreaticoduodenectomy.

### 3.3 Examination for fat deposition

Thirty‐four patients in the fat deposition group had a difference of ≥0.10. All 12 patients who had postoperative NAFLD were included in the fat deposition group. Pancreatic cancer was significantly more common in the fat deposition group than in the nonfat deposition group.

No significant differences in postoperative fat deposition by disease type were observed. Pancreatic leakage was significantly less frequent in the fat deposition group than in the nonfat deposition group (2.9% vs. 19.4%, *p* = 0.024). The time‐lapse until drain removal was 11 days in the nonfat deposition group and 6.8 days in the fat deposition group; however, the difference was not statistically significant (*p* = 0.1). Postoperative HbA1c was significantly lower in the fat deposition group than in the nonfat deposition group (5.8 vs. 6.3, *p* = 0.013). A significant decrease in postoperative BMI was observed in the fat deposition group (19.1 vs. 21.4, *p* = 0.002). Multivariate analysis revealed that a lower postoperative BMI was a risk factor for fat deposition (HR 0.56, 95% CI 0.523–0.982, *p* = 0.042). Multivariate analysis further revealed that the fat deposition group had significantly lower pancreatic leakage (HR 7.944, 95% CI 1.993–63.562, *p* = 0.049) and postoperative BMI (HR 0.56, 95% CI 0.523–0.982, *p* = 0.042) than the nonfat deposition group (Table 3).

**TABLE 3:** Comparison of clinical data between the fat deposition and nonfat deposition groups.

	Fat deposition (*n* = 34)	Nonfat deposition (*n* = 67)	Univariate analysis	Multivariate analysis		
			*p* value	HR	95% CI	*p* value
Sex (male:female)	14:20	43:24	0.54			NS
Age (year), mean (SD)	69.5 (11.0)	73.1 (8.0)	0.24			NS
Surgery duration (min), mean (SD)	214.8 (58.6)	241.5 (74.0)	0.054			NS
Blood loss volume (mL), mean (SD)	531.1 (556)	681.7 (578.4)	0.126			NS
Diagnosis						
Pancreatic carcinoma: other cancers	19:15	26:41	0.1			NS
Preoperative albumin (g/dL), mean (SD)	3.7 (0.6)	3.6 (0.5)	0.6			NS
Preoperative total protein (g/dL), mean (SD)	7.1 (0.8)	7.1 (0.9)	0.771			NS
Preoperative HbA1c (%), mean (SD)	6.3 (1.0)	6.6 (1.3)	0.2689			NS
Preoperative BMI, mean (SD)	21.5 (3.2)	22.8 (2.9)	0.07			NS
Surgical procedure						
SSPPD	27	56	0.65			NS
SSPPD + PV	2	7	0.58			NS
Lap‐SSPPD	3	3	0.254			NS
PD	1	1	0.352			NS
SSPPD + hepatectomy	1	0	0.4			NS
Postoperative albumin (g/dL), mean (SD)	3.5 (0.8)	3.7 (0.6)	0.189			NS
Postoperative total protein (g/dL), mean (SD)	6.9 (0.8)	7.1 (0.6)	0.45			NS
Postoperative HbA1c (%), mean (SD)	5.8 (0.4)	6.3 (0.8)	0.013			NS
Postoperative BMI, mean (SD)	19.1 (2.9)	21.4 (2.5)	0.002	0.56	0.523–0.982	0.042
Complications						
POPF ≥ Grade B	1 (2.9%)	13 (19.4%)	0.024	7.944	1.993–63.562	0.049
Bile leakage	6	2	0.082			NS
Postoperative bleeding	1	0	0.569			NS
SSI	1	0	0.569			NS
Pneumonia	0	1	0.448			NS
Anastomotic stenosis	0	1	0.448			NS
Days until drain removal, mean (SD)	6.8 (9.4)	11 (13.5)	0.1			NS
Hospital stay (days), mean (SD)	12.7 (11.0)	11.6 (6.1)	0.611			NS

Abbreviations: BMI, body mass index; CI, confidence interval; HR, hazard ratio; Lap‐SSPPD, laparoscopic SSPPD; NS, not significant; PD, pancreaticoduodenectomy; POPF, postoperative pancreatic fistula; PV, portal vein resection; SD, standard deviation; SSI, surgical site infection; SSPPD, subtotal stomach‐preserving pancreaticoduodenectomy.

## 4 DISCUSSION

In the present study, we investigated postoperative NAFLD and fat deposition after PD and identified pancreatic cancer and low postoperative BMI as risk factors for postoperative NAFLD. We also found that patients with postoperative fatty deposits had a low incidence of pancreatic leakage and lower postoperative BMIs.

The main difference between post‐PD NAFLD and the usual NAFLD is that the former occurs without insulin resistance,^6^ a phenomenon for which several mechanisms have been postulated. The first mechanism is impaired pancreatic exocrine function, which has been reported in 65.5% of PD cases.^16^ In pancreatic cancer, the lesion obstructs the pancreatic duct, causing caudal chronic pancreatitis and associated pancreatic atrophy, which in turn leads to decreased pancreatic exocrine function. Impaired pancreatic exocrine function results in fatty stools, and impaired fat absorption enhances the conversion of carbohydrates to fat in the liver.^9^ Neurogenic diarrhea associated with superior mesenteric artery (SMA) plexus dissection may also cause fat malabsorption and exacerbate NAFLD.^9^ The second possible mechanism is the presence of endotoxins that induce hepatic dysfunction. Intractable diarrhea caused by SMA plexus dissection or impaired pancreatic exocrine function presumably induces bacterial translocation due to intestinal mucosal atrophy. Endotoxins may enter the liver via the portal vein, activating Kupffer cells and inducing fatty deposition in the liver.^6^, ^9^, ^10^, ^17^ Cholangitis^18^ and postoperative infections^10^ have also been reported to induce the above mechanism. Thus, appropriate postoperative infection management is important for NAFLD prevention. Third, zinc deficiency may also cause NAFLD. Zinc binds to zinc‐binding proteins present in pancreatic secretions and is absorbed from the duodenum and proximal jejunum.^19^ Zinc deficiency causes diarrhea induced by intestinal mucosal atrophy. Furthermore, after extensive pancreatectomy, zinc concentrations in the blood and pancreatic tissues are markedly reduced, further impairing insulin secretion and pancreatic enzyme secretion.^20^ The incidence of zinc deficiency after PD, in which a portion of the duodenum and proximal jejunum is resected, has been reported to be 68%.^21^ Therefore, after PD, malnutrition develops in close association with exocrine dysfunction, diarrhea, infection, and zinc deficiency, resulting in NAFLD. In our study, pancreatic cancer was significantly more frequently observed in the NAFLD group compared to the non‐NAFLD group. Additionally, pancreatic leakage was significantly more frequently observed in the fat deposition group compared to the nonfat deposition group. Although not significant, no cases of pancreatic exocrine leakage were observed in the NAFLD group, suggesting that the pancreatic exocrine function was impaired; thus, pancreatic leakage did not occur. Hence, the above findings imply that decreased pancreatic exocrine function is associated with the pathogenesis of fat deposition. Patients in the NAFLD and fat deposition groups had lower postoperative BMIs, suggesting that impaired fat digestion and absorption due to impaired pancreatic exocrine function was the underlying cause and was significantly involved in the pathogenesis of hepatic fat deposition. All 12 patients who developed NAFLD after PD were included in the fat deposition group. Therefore, the new concept of fat deposition that we proposed in this study definitely reflects the process of postoperative NAFLD transition after PD.

Moore et al.^22^ reported that the incidence of pancreatic exocrine insufficiency after pancreatectomy varied depending on the surgical technique used, with PD, DP, and central resection accounting for 43%, 12%, and 5% of cases, respectively. In case of pancreatic head tumors, caudal pancreatic atrophy occurs; however, in most cases, the pancreatic tissue in the head of the organ is normal during caudal pancreatectomy and pancreatic function is thought to be preserved. Pancrelipase delayed‐release capsules (CREON), as a treatment option for pancreatic exocrine insufficiency, has been reported to be effective in decreasing the frequency of defecation, improving the absorption rate of fat and increasing the post‐pancreatectomy body weight.^23^, ^24^ Improvements in NAFLD after pancreatectomy have been reported to contribute to the prognosis of patients,^25^ especially those with pancreatic cancer.^26^ Our study suggests that CREON should be administered prophylactically to patients with pancreatic cancer after PD. In our study, pancreatic cancer was also a risk factor for NAFLD, and CREON should be administered after PD for pancreatic cancer. In addition, patients who do not develop pancreatic leakage may have impaired pancreatic exocrine function, and CREON administration may be considered as a NAFLD prophylaxis in such patients.

This study has several limitations. First, this was a single‐center retrospective study; therefore, its relevance may be considered primarily exploratory. Hence, a large‐scale multicenter study is desirable to demonstrate the reproducibility of the results of this study. Second, NAFLD is a quickly changing pathology that depends on the patient's condition and the passage of time. In our study, we used CT values obtained 6 months after PD; however, study data may vary if the period changes. Third, parameters such as pancreatic exocrine function and zinc levels were not evaluated. Future studies are needed to evaluate the post‐PD pancreatic exocrine function and clarify its relationship with fat deposition.

In conclusion, our findings suggest that postoperative BMI and pancreatic cancer are associated with a higher risk of postoperative NAFLD after PD, which may result from pancreatic exocrine insufficiency with impaired fat absorption.

## AUTHOR CONTRIBUTIONS

HI, HY, RF, ST, MM, EN, and HM performed the surgical procedures and postoperative management.

## FUNDING INFORMATION

Not available.

## CONFLICT OF INTEREST STATEMENT

The authors declare no conflicts of interest for this article.

## ETHICAL APPROVAL

The protocol for this research project was approved by a suitably constituted ethics committee of Tokai University in November 2022, approval no. 22R‐186, and the study was conducted per the principles of the Declaration of Helsinki. Informed consent was obtained from all participants or their guardians.

## CONSENT

Written informed consent was obtained from the patients for the publication of their personal data.

A copy of the written consent form is available for review from the Editor‐in‐Chief of this journal.

## References

[1] Watanabe S , Hashimoto E , Ikejima K , Uto H , Ono M , Sumida Y , et al. Evidence‐based clinical practice guidelines for nonalcoholic fatty liver disease/nonalcoholic steatohepatitis. J Gastroenterol. 2015;50(4):364–77. 25708290 10.1007/s00535-015-1050-7

[2] Ludwig J , Viggiano TR , McGill DB , Oh BJ . Nonalcoholic steatohepatitis: Mayo Clinic experiences with a hitherto unnamed disease. Mayo Clin Proc. 1980;55(7):434–8. 7382552

[3] Merriman RB , Aouizerat BE , Bass NM . Genetic influences in nonalcoholic fatty liver disease. J Clin Gastroenterol. 2006;40(Suppl 1):S30–3. 16540764 10.1097/01.mcg.0000168643.16074.19

[4] Huddy JR , Macharg FM , Lawn AM , Preston SR . Exocrine pancreatic insufficiency following esophagectomy. Dis Esophagus. 2013;26(6):594–7. 23199208 10.1111/dote.12004

[5] Sato T , Matsuo Y , Shiga K , Morimoto M , Miyai H , Takeyama H . Factors that predict the occurrence of and recovery from non‐alcoholic fatty liver disease after pancreatoduodenectomy. Surgery. 2016;160(2):318–30. 27211602 10.1016/j.surg.2016.04.009

[6] Tanaka N , Horiuchi A , Yokoyama T , Kaneko G , Horigome N , Yamaura T , et al. Clinical characteristics of de novo nonalcoholic fatty liver disease following pancreaticoduodenectomy. J Gastroenterol. 2011;46(6):758–68. 21267748 10.1007/s00535-011-0370-5

[7] Fujii Y , Nanashima A , Hiyoshi M , Imamura N , Yano K , Hamada T . Risk factors for development of nonalcoholic fatty liver disease after pancreatoduodenectomy. Ann Gastroenterol Surg. 2017;1(3):226–31. 29863141 10.1002/ags3.12024 PMC5881353

[8] Luu C , Thapa R , Rose T , Woo K , Jeong D , Thomas K , et al. Identification of nonalcoholic fatty liver disease following pancreatectomy for noninvasive intraductal papillary mucinous neoplasm. Int J Surg. 2018;58:46–9. 30218781 10.1016/j.ijsu.2018.09.002 PMC7771269

[9] Kato H , Isaji S , Azumi Y , Kishiwada M , Hamada T , Mizuno S , et al. Development of nonalcoholic fatty liver disease (NAFLD) and nonalcoholic steatohepatitis (NASH) after pancreaticoduodenectomy: proposal of a postoperative NAFLD scoring system. J Hepatobiliary Pancreat Sci. 2010;17(3):296–304. 19809782 10.1007/s00534-009-0187-2

[10] Sato R , Kishiwada M , Kuriyama N , Azumi Y , Mizuno S , Usui M , et al. Paradoxical impact of the remnant pancreatic volume and infectious complications on the development of nonalcoholic fatty liver disease after pancreaticoduodenectomy. J Hepatobiliary Pancreat Sci. 2014;21(8):562–72. 24824077 10.1002/jhbp.115

[11] Nakagawa N , Murakami Y , Uemura K , Sudo T , Hashimoto Y , Kondo N , et al. Nonalcoholic fatty liver disease after pancreatoduodenectomy is closely associated with postoperative pancreatic exocrine insufficiency. J Surg Oncol. 2014;110(6):720–6. 24965234 10.1002/jso.23693

[12] Song SC , Choi SH , Choi DW , Heo JS , Kim WS , Kim MJ . Potential risk factors for nonalcoholic steatohepatitis related to pancreatic secretions following pancreaticoduodenectomy. World J Gastroenterol. 2011;17(32):3716–23. 21990953 10.3748/wjg.v17.i32.3716 PMC3181457

[13] Child CG . Pancreaticojejunostomy and other problems associated with the surgical management of carcinoma involving the head of the pancreas: report of five additional cases of radical pancreaticoduodenectomy. Ann Surg. 1944;119(6):845–55. 17858411 10.1097/00000658-194406000-00004 PMC1617983

[14] Ricci C , Longo R , Gioulis E , Bosco M , Pollesello P , Masutti F , et al. Noninvasive in vivo quantitative assessment of fat content in human liver. J Hepatol. 1997;27(1):108–13. 9252082 10.1016/s0168-8278(97)80288-7

[15] Bassi C , Marchegiani G , Dervenis C , Sarr M , Abu Hilal M , Adham M , et al. The 2016 update of the international study group (ISGPS) definition and grading of postoperative pancreatic fistula: 11 years after. Surgery. 2017;161(3):584–91. 28040257 10.1016/j.surg.2016.11.014

[16] Okano K , Murakami Y , Nakagawa N , Uemura K , Sudo T , Hashimoto Y , et al. Remnant pancreatic parenchymal volume predicts postoperative pancreatic exocrine insufficiency after pancreatectomy. Surgery. 2016;159(3):885–92. 26603844 10.1016/j.surg.2015.08.046

[17] Murata Y , Mizuno S , Kato H , Kishiwada M , Ohsawa I , Hamada T , et al. Nonalcoholic steatohepatitis (NASH) after pancreaticoduodenectomy: association of pancreatic exocrine deficiency and infection. Clin J Gastroenterol. 2011;4(4):242–8. 26189528 10.1007/s12328-011-0226-9

[18] Tilg H , Moschen AR . Evolution of inflammation in nonalcoholic fatty liver disease: the multiple parallel hits hypothesis. Hepatology. 2010;52(5):1836–46. 21038418 10.1002/hep.24001

[19] Evans GW . Normal and abnormal zinc absorption in man and animals: the tryptophan connection. Nutr Rev. 1980;38(4):137–41. 7207876 10.1111/j.1753-4887.1980.tb05874.x

[20] Kato K , Isaji S , Kawarada Y , Hibasami H , Nakashima K . Effect of zinc administration on pancreatic regeneration after 80% pancreatectomy. Pancreas. 1997;14(2):158–65. 9057188 10.1097/00006676-199703000-00008

[21] Yu HH , Yang TM , Shan YS , Lin PW . Zinc deficiency in patients undergoing pancreatoduodenectomy for periampullary tumors is associated with pancreatic exocrine insufficiency. World J Surg. 2011;35(9):2110–7. 21691869 10.1007/s00268-011-1170-z

[22] Moore JV , Tom S , Scoggins CR , Philips P , Egger ME , Martin RCG 2nd . Exocrine pancreatic insufficiency after pancreatectomy for malignancy: systematic review and optimal management recommendations. J Gastrointest Surg. 2021;25(9):2317–27. 33483914 10.1007/s11605-020-04883-1

[23] Gubergrits N , Malecka‐Panas E , Lehman GA , Vasileva G , Shen Y , Sander‐Struckmeier S , et al. A 6‐month, open‐label clinical trial of pancrelipase delayed‐release capsules (Creon) in patients with exocrine pancreatic insufficiency due to chronic pancreatitis or pancreatic surgery. Aliment Phramacol Ther. 2011;33(10):1152–61. 10.1111/j.1365-2036.2011.04631.x 21418260

[24] Whitcomb DC , Lehman GA , Vasileva G , Malecka‐Panas E , Gubergrits N , Shen Y , et al. Pancrelipase delayed‐release capsules (CREON) for exocrine pancreatic insufficiency due to chronic pancreatitis or pancreatic surgery: a double‐blind randomized trial. Am J Gastroenterol. 2010;105(10):2276–86. 20502447 10.1038/ajg.2010.201

[25] Okamura Y , Sugimoto H , Yamada S , Fujii T , Nomoto S , Takeda S , et al. Risk factors for hepatic steatosis after pancreatectomy: a retrospective observational cohort study of the importance of nutritional management. Pancreas. 2012;41(7):1067–72. 22617712 10.1097/MPA.0b013e31824c10ab

[26] Kanda M , Fujii T , Kodera Y , Nagai S , Takeda S , Nakao A . Nutritional predictors of postoperative outcome in pancreatic cancer. Br J Surg. 2011;98(2):268–74. 20960457 10.1002/bjs.7305

